# Principles and practice of designing microbial biocatalysts for fuel and chemical production

**DOI:** 10.1093/jimb/kuab016

**Published:** 2021-03-04

**Authors:** K T Shanmugam, Lonnie O Ingram

**Affiliations:** Department of Microbiology and Cell Science, University of Florida, Gainesville, FL 32611, USA; Department of Microbiology and Cell Science, University of Florida, Gainesville, FL 32611, USA

**Keywords:** Fermentation, Metabolic engineering, Primary products, Biofuel, Physiology

## Abstract

The finite nature of fossil fuels and the environmental impact of its use have raised interest in alternate renewable energy sources. Specifically, nonfood carbohydrates, such as lignocellulosic biomass, can be used to produce next generation biofuels, including cellulosic ethanol and other nonethanol fuels like butanol. However, currently there is no native microorganism that can ferment all lignocellulosic sugars to fuel molecules. Thus, research is focused on engineering improved microbial biocatalysts for production of liquid fuels at high productivity, titer, and yield. A clear understanding and application of the basic principles of microbial physiology and biochemistry are crucial to achieve this goal. In this review, we present and discuss the construction of microbial biocatalysts that integrate these principles with ethanol-producing *Escherichia coli* as an example of metabolic engineering. These principles also apply to fermentation of lignocellulosic sugars to other chemicals that are currently produced from petroleum.

Availability of inexpensive fossil fuels, such as petroleum, natural gas, and coal, has positively transformed human culture and the world economy. However, the rate of increase in the use of this limited resource by an expanding population is causing environmental changes at the global level (National Research Council, 2010). In addition, future energy shortage would have devastating consequences on the quality of life. Thus, it is imperative that fossil fuels are supplemented with alternate, renewable sources of energy to extend the lifespan of this dwindling reserve (Fig. 1). Since more than two-third of the petroleum is used in transportation, a renewable source of liquid fuel is urgently needed.

**Fig. 1.**
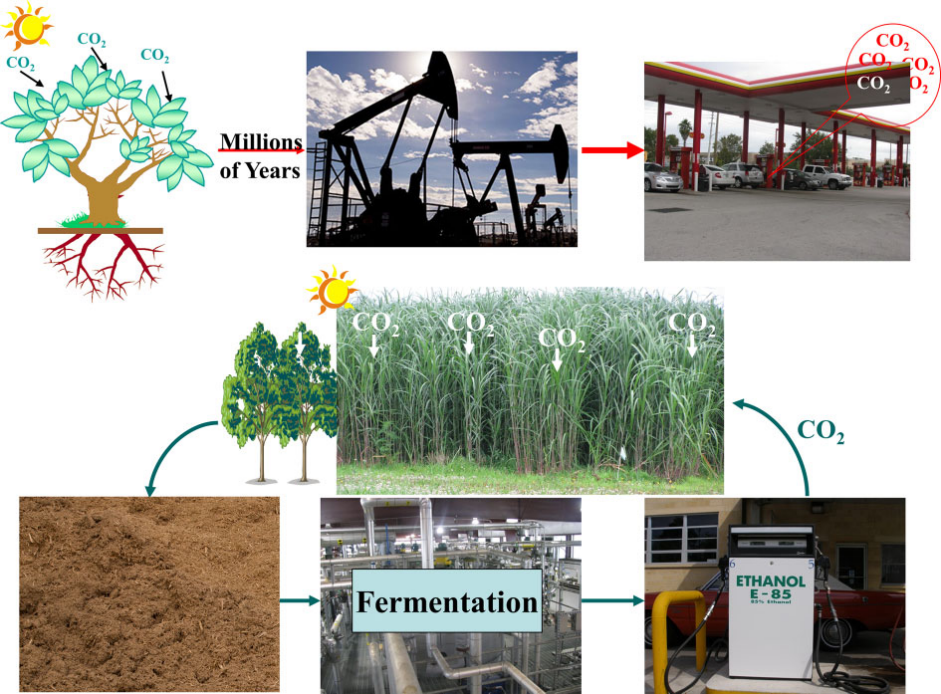
Fossil fuel-based linear conversion of biomass carbon to energy and CO_2_ (top), and cyclic energy production/utilization with CO_2_ as solar energy carrier (bottom). (Top panel) Although the process of photosynthetic conversion of CO_2_ to biomass and release of this fixed CO_2_ during the delivery of stored energy is cyclic, separation of the two halves of the process by millions of years, makes this production/consumption linear in a geological time scale. (Bottom panel) Cyclic CO_2_ capture and release during solar energy conversion process. Picture of oil wells: https://www.niehs.nih.gov/health/topics/agents/fracking/index.cfm.

Ethanol, currently produced from hexose-rich food and feed crops using the yeast *Saccharomyces cerevisiae*, is used as a renewable fuel, fuel additive, and extender (Giebel et al., 2011). Although this has reduced petroleum use, it comes at the cost of a competition between food and fuel, which will even intensify in the coming years owing to population growth (Tenenbaum, 2008). Thus, alternative carbohydrate sources such as lignocellulosic biomass along with increased acreage to cultivate them must be developed. Lignocellulosic biomass-based biofuels are expected to be C-neutral since the CO_2_ released during energy extraction from fuel molecules is fixed by the plants, recovered as carbohydrates and fermented by microorganisms to energy rich fuel compounds in a cyclic manner (Fig. 1, bottom). This process replaces the millions of years of fossilization of biomass into petroleum that is extracted from the ground and used today as liquid fuels in a linear fashion resulting in CO_2_ release into the atmosphere. In this process, the CO_2_ consumption and release are separated by millions of years (Fig. 1, top) leading to negative environmental impact.

Microorganisms are required for bioconversion of nonfood carbohydrates and they should be able to hydrolyze complex carbohydrates (using glycan hydrolases), ferment diverse soluble saccharides and sugars (hexoses, pentoses, uronic acids, and aminosugars), ferment gases (CO, H_2_) to higher molecular weight products, and resist inhibitors generated during chemical processing of biomass (Fig. 2). To date, no microorganism has been isolated from nature that produces liquid fuels from all the sugars in nonfood crops with the selectivity, productivity, titer, and yield needed to be commercially viable. Therefore, development of engineered microbial biocatalysts is paramount to future renewable fuel production (reviewed in Alper & Stephanopoulos, 2009; Gonzalez, 2013; Liao et al., 2016; Peralta-Yahya et al., 2012). New microbial biocatalysts are being developed using synthetic biology approaches, mainly by genome reconstruction of platform organisms like *Escherichia coli* and *S. cerevisiae* using synthetic parts. This can be simple genetic alterations that change the metabolism to total genome scale engineering as in the case of a synthetic cell (Gibson et al., 2008; Gonzalez, 2013; Nielsen & Keasling, 2016; Philp et al., 2013; Shakeel et al., 2019). *E. coli* and *S. cerevisiae* are preferred for genetic and metabolic engineering toward biofuel production due to the ease of genetic manipulation and the availability of large amount of physiological information.

**Fig. 2.**
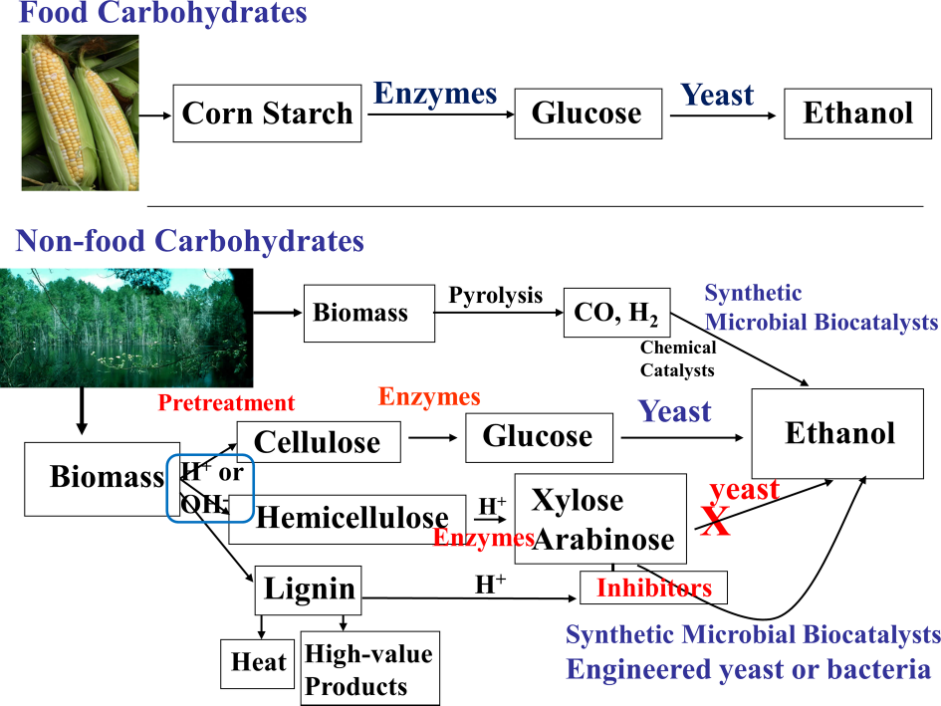
Biochemical processes for conversion of food (top) and nonfood carbohydrates to ethanol (bottom). Corn-derived starch is hydrolyzed to glucose by enzymes and the sugar is fermented to ethanol by yeast in a well-established process. Although several processes have been developed, conversion of lignocellulosic biomass to fermentable sugars is more complex than corn to glucose conversion. About 70% of biomass (by weight) can be converted to two types of fermentable sugars, hexoses and pentoses. Since wild type *S. cerevisiae* lacks the ability to ferment pentoses (Scalcinati et al., 2012), a new class of homoethanol-producing microbial biocatalysts are needed for pentose fermentation in the presence of inhibitors. These microorganisms, both native and metabolically engineered, are expected to produce a variety of chemicals as petroleum supplements and replacements. Pyrolysis of biomass to CO and H_2_ followed by catalytic or microbial conversion of CO and H_2_ is another biomass to liquid fuel process that is not discussed in this review.

*S. cerevisiae* dominates alcoholic fermentation of food and feed carbohydrates owing to its specific physiological characteristics (Table 1) (Piskur et al., 2006) and these characteristics are also desired in any new microbial biocatalyst that is developed for production of cellulosic ethanol and other fuels. However, a significant fraction of “nonfood” biomass-derived sugars are pentoses which are poorly fermented by *S. cerevisiae* (Scalcinati et al., 2012).

**Table 1.** Physiological Characteristics that are Desired in a Microbial Biocatalyst for Fermentation of Biomass-Derived Sugars to Fuels

I. Properties that make *S. cerevisiae* a choice microbial biocatalyst for ethanol production
1. High growth rate
2. High cell yield
3. High productivity
Specific and volumetric
4. High product titer
5. High selectivity
6. Preference for fermentative metabolism even when O_2_ is available
7. High ethanol tolerance
8. Flocculation or rapid settlement for easy removal from broth
9 Hardiness (aerobic, anaerobic, low pH, desiccation, storage)
10. Relative ease of genetic manipulation (for a eukaryote)
11. Wealth of fundamental biochemical, physiological, and genetic information
12. Extensive history of commercial successes
**II. Additional desired characteristics of a microbial biocatalyst for fermentation of biomass**
1. Minimal growth requirements
2. Metabolic versatility
3. Co-utilization of various sugars
4. Tolerate high sugar concentration
5. Resistance to inhibitors
6. Insensitive to product inhibition
7. High-value co-products
8. Amenable to genetic engineering
9. Produce glycan hydrolases (such as cellulases and xylanases)

In addition, ethanol is not considered as an ideal biofuel due to its low energy density compared to gasoline (∼55%; 25 MJ/kg compared to 44–46 MJ/kg of gasoline) and its corrosive nature. To overcome this limitation, fermentation of sugars to next-generation liquid fuels (butanol, alkanes, fatty acid esters, etc.) requires a new class of microbial biocatalysts that, in addition to the desired physiological characteristics of yeast, have properties required for cost-effective production of these chemicals. In this review, the principles of microbial physiology that govern the production of chemicals as primary products of anaerobic metabolism at high titer and yield are discussed. The aim is to provide a physiology-based roadmap for designing and developing microbial biocatalysts with ethanol as the model product due to its simplicity. These basic tenets apply to the design and construction of microbial biocatalysts for production of other next-generation fuels at high titer, yield, and productivity.

## Carbon-Based Energy

**Fig. 3.**
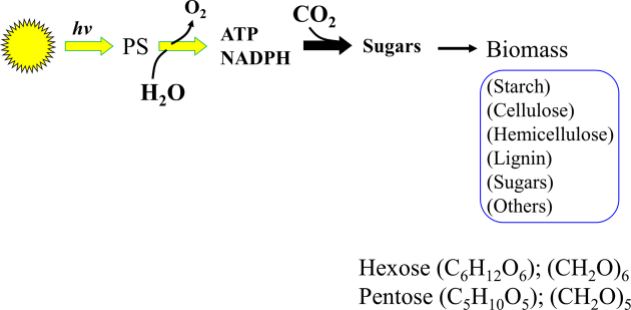
Reduction of CO_2_ to biomass for production of organic energy reserves using solar energy and H_2_O. PS, photosynthesis.

**Fig. 4.**
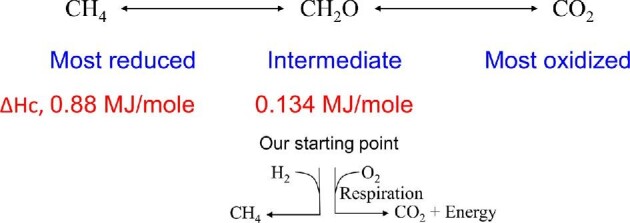
Oxidation states of carbon. Methane is the most reduced form with CO_2_ as the most oxidized form of carbon. Glucose (CH_2_O)_6_, the common sugar substrate, is at the intermediate redox state.

 In 2017, 85% of energy consumed in the United States of America was derived from oxidation of carbon to CO_2_. Of this total, fossil fuel sources (petroleum, natural gas, and coal) contributed 80% and renewable fuels (biofuels, wood, plant waste, etc.) provided only 5% of the total (EIA-DOE). These energy reserves (fossil or renewable) are derivatives of photosynthesis that capture atmospheric CO_2_ and reduce it to various redox states utilizing water and solar energy (Fig. 3). Carbon exists in various states of reduction/oxidation (redox) with CH_4_ as the reduced form and CO_2_ as the oxidized form, and the energy content of a given compound depends on C-length and average redox state of the carbon (Fig. 4 and Table 2). Biomass, the primary feedstock of renewable energy is rich in oxygen and thus has significantly lower energy content compared to fossil fuels with less than 1% oxygen content. For example, glucose (C_6_H_12_O_6_), the common organic energy source used by biological systems, releases 2,81 MJ/mol energy upon complete combustion to 6 CO_2_ (Δ*H*_c_) and the presence of oxygen in the molecule reduces the energy content of glucose by about 33% compared to an analogous fully reduced six carbon compound, hexane (C_6_H_14_; 4.16 MJ/mol; Table 2) (Domalski, 1972). Due to this constraint, renewable energy production by fermentation starts with carbon at an average energy content and redox state of glucose, (CH_2_O)_6_. It should be noted that although hydrocarbons are produced by microorganisms by fermentation of glucose, the redox state of carbon in all products combined cannot exceed that of starting glucose-C. This dictates that for every product that is more reduced than the feed glucose, there is another co-product that is more oxidized than glucose in the final product profile of an anaerobic microorganism. In the fermentation of glucose to ethanol (Reaction 1), ethanol is more reduced (CH_3_O_0.5_)_2_ (redox state of −2) than glucose (CH_2_O)_6_ (redox state of 0) and this redox difference is offset by the co-product CO_2_ (redox state of +2) that is more oxidized than glucose.

(Reaction 1) \begin{eqnarray*} {{\rm{C}}_6}{{\rm{H}}_{12}}{{\rm{O }}_6} \to 2\,{{\rm{C}}_2}{{\rm{H}}_6}{\rm{O}} + 2\,{\rm{C}}{{\rm{O}}_2} \end{eqnarray*}

Although the yield of ethanol per glucose is two on a molar basis, it is only 0.51 on a weight basis (g product/g substrate consumed) due to the co-product, CO_2_. However, fermentation of glucose to ethanol (Δ*H*_c_ of −1.37 MJ/mol × 2) conserves about 97% of the energy in glucose (Δ*H*_c_, −2.81 MJ/mol) and is the most efficient conversion of energy from glucose to a fuel molecule to date. Other aliphatic alcohols such as butanol (Δ*H*_c_, −2.68 MJ/mol; Reaction 2) or hexanol (Δ*H*_c_, −3.985 MJ/mol; derived from 1.5 glucose; Reaction 3) retain about 94–95% of the energy in glucose during fermentation.

(Reaction 2) \begin{eqnarray*} {{\rm{C}}_6}{{\rm{H}}_{12}}{{\rm{O }}_6} \to {{\rm{C}}_4}{{\rm{H}}_{10}}{\rm{O}} + 2\,{\rm{C}}{{\rm{O}}_2} + {{\rm{H}}_2}{\rm{O}} \end{eqnarray*}

(Reaction 3) \begin{eqnarray*} 1.5\,{{\rm{C}}_6}{{\rm{H}}_{12}}{{ \rm{O}}_6} \to {{\rm{C}}_6}{{\rm{H}}_{14}}{\rm{O}} + 3\,{\rm{C}}{{\rm{O}}_2} + 2\,{{\rm{H}}_2}{\rm{O}} \end{eqnarray*}

**Table 2.** Energy Content Depends on the Chain Length and Oxidation State of Carbon

Compound	Formula	Δ*H*_c_^a^ (MJ/mol)	Δ*H*_c_^a^ (kcal/mol)
Methane	CH_4_	−0.89	−212.8
Ethane	C_2_H_6_	−1.56	−372.8
Propane	C_3_H_8_	−2.22	−530.6
Butane	C_4_H_10_	−2.88	−687.7
Pentane	C_5_H_12_	−3.51	−838.8
Hexane	C_6_H_14_	−4.16	−995.0
Ethanol	C_2_H_6_O	−1.37	−326.7
Ethanediol	C_2_H_6_O_2_	−1.18	−282.9
Acetaldehyde	C_2_H_4_O	−1.17	−278.8
Acetic acid	C_2_H_4_O_2_	−0.88	−209.0
Glucose	C_6_H_12_O_6_	−2.81	−671.4

^a^Heat of combustion (data from Domalski, 1972).

Due to the thermodynamic constraint that matter cannot be created or destroyed during a reaction, the redox state of the carbon in the substrates is also maintained in the total products. This requires that during fermentative production of biofuels in which the carbon is more reduced than the substrates, such as glucose, will always be fraught with low yield (mass) but not necessarily with low energy yield. This lower product yield on a mass basis (ethanol, butanol, and hexanol at 0.51, 0.41, and 0.38 g/g, respectively) is a result of anaerobic metabolic pathway limitations (Kang & Lee, 2015; Weusthuis et al., 2011). Any attempt to maintain high C-yield coupled with high energy content of the product is expected to require input of reductant such as H_2_ from outside to balance the overall redox stoichiometry. For example, glucose can be hydrogenated to sorbitol and hydrogenolysis of sorbitol can yield hexane at high yield with H_2_ as the reductant (Reaction 4) (Liu et al., 2014). This reaction is catalyzed by inorganic catalysts and not by microbial biocatalysts, again due to metabolic limitations in the conversion of sorbitol to hexane.

(Reaction 4) \begin{eqnarray*} {\rm{Glucose}}\stackrel{{{\rm{Catalyst}} + {{\rm{H}}_2}}}{-\!\!\!-\!\!\!-\!\!\!-\!\!\!-\!\!-\!\!\!\longrightarrow}{\rm{Sorbitol}}\stackrel{{{\rm{Catalyst}} +{{\rm{H}}_2}}}{-\!\!\!-\!\!\!-\!\!\!-\!\!\!-\!\!-\!\!\!\longrightarrow}{\rm{Hexane}} \end{eqnarray*}

## Maintaining Energy and Redox Balance

For construction of microbial biocatalysts toward production of renewable biofuels and chemicals, two physiological properties of the microorganism are critical: energy and redox balance. The energy and redox state of a cell determine microbial growth and subsequently product titer (g product/l) and productivity [specific productivity; g product/(g cell biomass hr); volumetric productivity; g product/(l hr)].

### Fuel Production in Aerobic Condition

In all living systems ATP is the energy currency and microbial growth is directly proportional to the net ATP generated during metabolism of a substrate. This can be measured as the cell yield (*Y*_ATP_), defined as the amount of cells produced per mole of ATP hydrolyzed to ADP and Pi, and is about 10.5 g dry biomass/mol ATP for *E. coli* and other bacteria (Payne, 1970; Tempest & Neijssel, 1984). Due to this relationship, cell yield is positively correlated with the ATP yield per metabolized carbon compound. An aerobic culture, compared to one growing anaerobically, produces more ATP per glucose owing to complete oxidation of substrate carbon to CO_2_, grows faster and produces more cell mass per C-source consumed (*Y*_glucose_) (Payne, 1970). However, potential biofuels and biochemicals are products of incomplete oxidation of sugar substrates and therefore aerobic conditions are not ideal for production of these chemicals at high titer and yield (g product/g of C-source consumed).

Various approaches are used to favor generation of incomplete oxidation products under aerobic condition: limiting O_2_ concentration, eliminating terminal oxidase by mutation, overexpressing appropriate metabolic pathway(s) toward the desired product, etc. (Portnoy et al., 2008). In these studies, production of NADH and oxidation of NADH to NAD^+^ with O_2_ are uncoupled. The higher NADH/NAD^+^ ratio observed during O_2_-limitation lowers the rate of production of acetyl-CoA by pyruvate dehydrogenase due to inhibition of the enzyme by NADH (Kim et al., 2008) and this leads to utilization of metabolically generated carbon compounds such as pyruvate as an electron acceptor to maintain appropriate redox balance (NADH/NAD^+^). This diversion of carbon to products results in lower ATP yield and cell growth with an associated increase in glycolytic flux to compensate for this reduction in ATP production (Koebmann, Westerhoff, Snoep, Nilsson, et al., 2002; Larsson et al., 1997). A native or an engineered synthetic pathway introduced to maintain redox balance under these conditions needs to be in synchrony with this change in glycolytic flux to accommodate the elevated pool of glycolysis intermediates and rapidly convert them to the desired product.

### Fuel Production Under Anaerobic Condition

During anaerobic growth, the ATP yield from glucose is limited and as *Y*_ATP_ is relatively constant for a given organism, the anaerobic cell yield per glucose fermented (*Y*_glucose_) is also lower compared to an aerobic culture (Daran-Lapujade et al., 2007; Gonzalez et al., 2017). For example, during fermentation of glucose to two molecules of ethanol (Reaction 1), 97% of the energy in glucose remains conserved in ethanol carbons (Δ*H*_c_ of 2.74 MJ out of 2.81 MJ/mol for glucose) and very little energy (∼70 kJ/mol glucose) is available to support growth. Taking a Δ*G*° value of −31.5 kJ/mol for ATP hydrolysis to ADP and inorganic phosphate (−7.3 kcal/mol) (Rosing & Slater, 1972), this leads to a maximum yield of 2 mol of ATP per mole of glucose fermented to ethanol. It should be noted that the energy yields of reactions are derived from heats of combustion of various compounds for this discussion and the net energy yields of reactions within an active dynamic cell differs significantly from these values depending on several factors, including the concentration of substrates and products. Due to this low ATP yield, the highest aerobic growth rate is not realized during anaerobic (fermentative) growth (0.6–0.7 vs. 0.33–0.38 per hr, respectively) (Gonzalez et al., 2017; McCloskey et al., 2014). The microorganism attempts to compensate for this lower than optimal ATP yield per glucose to pyruvate (Reaction 5) by increasing the glycolytic flux to elevate the rate of ATP production [8 vs. 13 mmol/(g cell dry weight hr), for aerobic and anaerobic condition, respectively] (Daran-Lapujade et al., 2007; Gonzalez et al., 2017; McCloskey et al., 2014).

(Reaction 5) \begin{eqnarray*} &&{\rm{Glucose}} + 2\,{\rm{ADP}} + 2\,{\rm{Pi}} + 2\,{\rm{NA}}{{\rm{D}}^ + } + 2\,{{\rm{H}}^ + }\\ &&\quad \to 2\,{\rm{Pyruvate}} + 2\,{\rm{ATP}} + 2\,{\rm{NADH}} \end{eqnarray*}

**Fig. 5.**
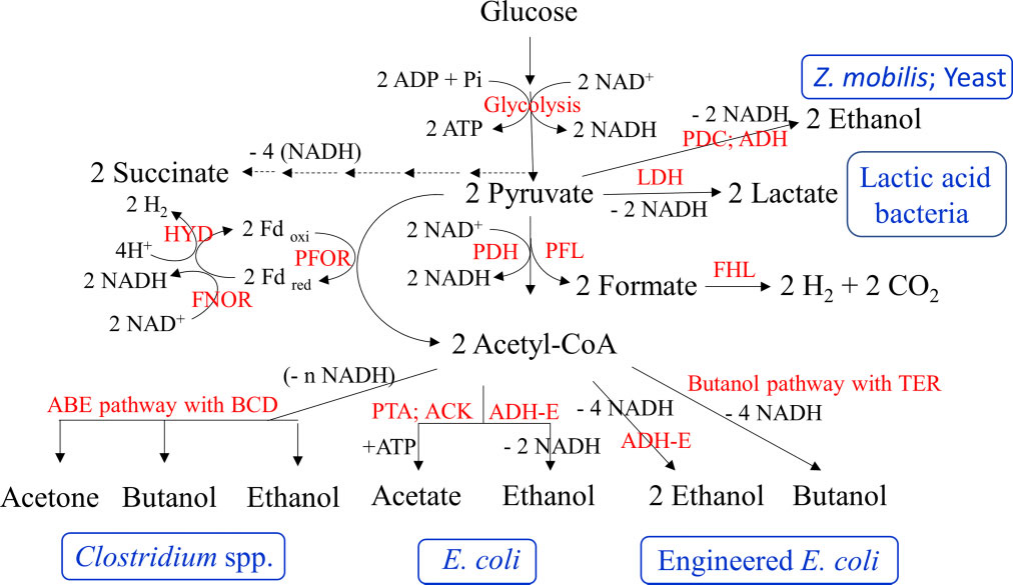
Fermentation pathways leading to production of primary products. ABE, acetone, butanol, ethanol; ACK, acetate kinase; ADH, alcohol dehydrogenase; ADH-E, aldehyde/alcohol dehydrogenase; BCD, butyryl-CoA dehydrogenase complex; FHL, formate-hydrogen-lyase; FNOR, ferredoxin-NADH oxidoreductase; HYD, hydrogenase; LDH, lactate dehydrogenase; PDC, pyruvate decarboxylase; PDH, pyruvate dehydrogenase; PFL, pyruvate-formate-lyase; PFOR, pyruvate-ferredoxin oxidoreductase; PTA, phosphotransacetylase; TER, trans-enoyl-CoA reductase.

The anaerobic glucose flux during active growth phase under this energy-starved state sets the maximum for the microorganism's glycolytic flux and the specific productivity of the desired product tends to maximize around this value. Part of the reductant produced during this process is used for biosynthesis and the remaining NADH is oxidized using pyruvate, the end product of glycolysis or compounds derived from pyruvate, such as acetyl-CoA, acetaldehyde, etc., to products that can serve as biofuels (such as ethanol or butanol) or green biochemicals (butyrate, lactate, acetone, butanediol, propanediol, etc.) (Fig. 5 and Table 3). These compounds are produced by the anaerobic cell to maintain redox balance for continued operation of glycolysis for ATP production to support growth and maintenance of the cell. It should be noted that production of these fermentation products is a result of an imbalance in the production and cellular demand of ATP and NADH during glycolysis.

**Table 3.** Redox Balance of Fermentation Product Profiles of Selected Bacteria

		Mole/mole of glucose fermented			
Product	O/R value	*E. coli*	*B. coagulans*	*C. acetobutylicum*	*Z. mobilis*
H_2_	−1	0.75		1.35	
CO_2_	+2	0.88		2.21	1.93
Formate	+1	0.02			
Acetate	0	0.37	0.04	0.14	0.01
Ethanol	−2	0.50	0.07	0.07	1.89
Lactate	0	0.80	1.77		
Succinate	+1	0.11			
Acetone	−2			0.22	
Butyrate	−2			0.04	
Butanol	−4			0.56	
Acetoin	−2				0.02
Carbon recovery		0.91	0.92	0.95	0.96
O/R balance		1.08	∼1.0	1.05	1.01

*Note*. Average O/R value for glucose (CH_2_O)_6_ is set at zero and the O/R values for various products were calculated based on the average oxidation state of carbon with reference to glucose. Carbon recovery values represent the amount of carbon in fermentation products and did not include the carbon in biomass. O/R balance value for *B. coagulans* that produced lactate as the major fermentation product (95% of total) was not calculated since the only reduced product, ethanol, was less than 4% of total products. Values for *E. coli, B. coagulans* and *C. acetobutylicum* are from Ingraham et al. (1983), Wang, Ingram and Shanmugam (2011), and Jones and Woods (1986), respectively. Values for *Z. mobilis* are from Kim et al. (2000) and the CO_2_ value was calculated from the product profile.

In a simple process, a synthetic lactate-producing *E. coli* (Grabar et al., 2006; Shanmugam & Ingram, 2008) uses pyruvate, the end product of glycolysis, as an electron acceptor (from NADH), which results in lactate production and secretion into the medium (Reaction 6).

(Reaction 6) \begin{eqnarray*} {{\rm{C}}_6}{{\rm{H}}_{12}}{{\rm{O }}_6} + 2\,{\rm{ADP}} + 2\,{\rm{Pi}} \to 2\,{{\rm{C}}_3}{{\rm{H}}_6}{{\rm{O}}_3} + 2\,{\rm{ATP}} \end{eqnarray*}

Net ATP and NADH production during the conversion of glucose to 2 pyruvates by the EMP-pathway is two each (Reaction 5) and further reduction of pyruvate to lactate yields 2 net ATPs while maintaining NADH/NAD^+^ ratio at an optimal level (Reaction 6; Fig. 5). The amount of ATP needed for production of 1 g of microbial cell can be calculated from *Y*_ATP_ values and other fermentation data, and this is about 100–120 mmol of ATP per gram of dry cell mass produced (Payne, 1970; Stouthamer & Bettenhaussen, 1977; Verduyn et al., 1991; Wang et al., 2019). Although all the ATP produced by the cell is consumed to support growth, maintenance and for other thermodynamic considerations that support reaction kinetics (released as heat), cell growth only consumes about 20–25% of the co-generated reductant. The remaining reductant needs to be oxidized to maintain redox balance, a prerequisite for continued generation of ATP. This is the primary reason for production of fermentation products by a microorganism; imbalance in the amount of ATP and reductant consumed during cell growth and the need for redox balance.

## Primary and Secondary Products

Products produced by microorganisms can be grouped into two broad categories: primary and secondary. The primary products are produced during anaerobic or O_2_-limited growth of heterotrophic microorganisms and are associated with maintaining redox balance to support glycolysis for ATP production (fermentation products; Fig. 5). Production of these chemicals are coupled to anaerobic cell growth and mutants that lack the ability to produce these chemicals do not grow anaerobically (Hasona et al., 2004; Kim et al., 2007; Mat-Jan et al., 1989; Wang, Ingram & Shanmugam, 2011). Some of the examples of primary products are ethanol production by *S. cerevisiae* and *Zymomonas mobilis*, lactate production by lactic acid bacteria, acetone, butanol, and ethanol by Clostridia, as well as various organic acids, such as propionate, butyrate, etc., by several microorganisms. Specific productivity of this group of chemicals is always the highest during the exponential phase of growth (the highest energy need) and declines with declining growth rate at late-exponential and stationary phase of growth (Tao et al., 2001; Wang et al., 2015). Ethanol or lactate is produced by these microorganisms at high yield; >95% of glucose carbon can be recovered in products. Several microorganisms produce a mixture of primary products and the ratio of these products in the culture medium is always balanced to maintain redox balance while meeting the energy demand of the organism (Table 3 and Fig. 5). As an example, wild-type *E. coli* produces acetate, ethanol, and formate as primary products during active growth at which an ATP/glucose yield of 3 can be supported by the actively growing, energy-starved culture (Reaction 7).

(Reaction 7) \begin{eqnarray*} {{\rm{C}}_6}{{\rm{H}}_{12}}{{\rm{O }}_6} + {{\rm{H}}_2}{\rm{O}} \to {{\rm{C}}_2}{{\rm{H}}_4}{{\rm{O}}_2} + {{\rm{C}}_2}{{\rm{H}}_6}{\rm{O}} + 2\,{\rm{C}}{{\rm{H}}_2}{{\rm{O}}_2}\left( {{\rm{Net\;ATP}}\,{\rm{yield}}:3} \right)\\ \end{eqnarray*}

(In this reaction, the extra oxygen comes from the reaction of phosphotransacetylase and acetate kinase. Water, instead of phosphate, is used here to simplify the equation.)

**Fig. 6.**
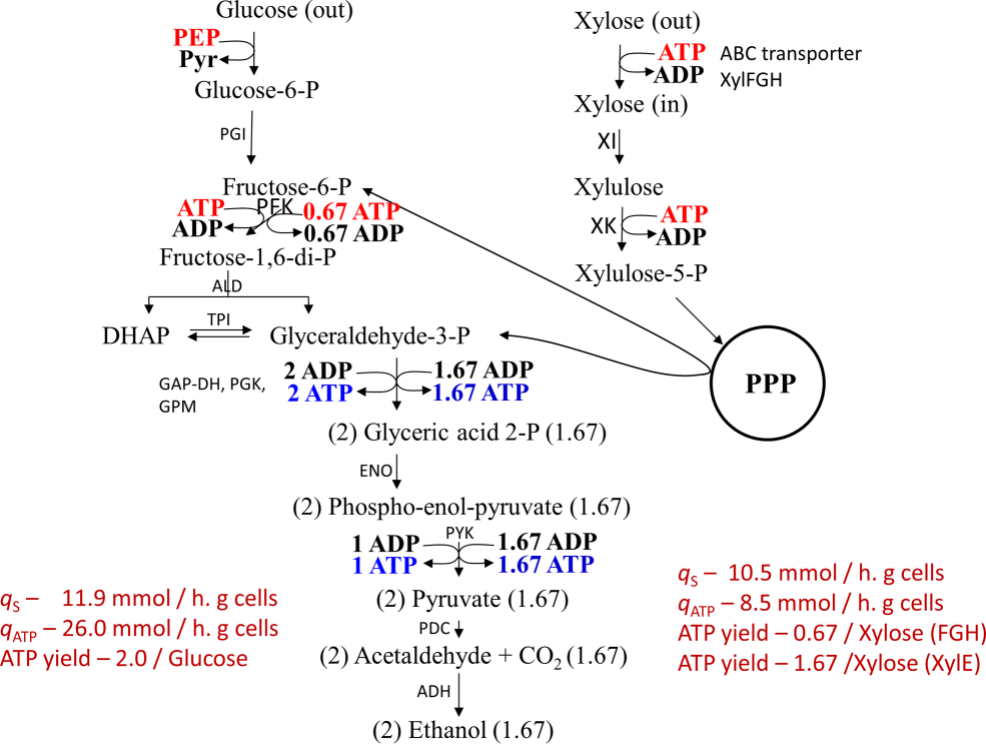
Glucose and xylose fermentation pathways in ethanologenic *E. coli* with different ATP yields. Numbers on the left side of the arrows and right side of the arrows represent the ATP yields for glucose and xylose, respectively. ALD, aldolase; ENO, enolase; GAP-DH, glyceraldehyde-3-phosphate dehydrogenase; GPM, 2,3-bisphosphoglycerate mutase; PFK, phosphofructokinase; PGI, glucose-6-phosphate isomerase; PGK, phosphoglycerate kinase; PYK, pyruvate kinase; TPI, triose-phosphate isomerase; XI, xylose isomerase; XK, xylulose kinase; XYL-E, xylose symport; XYL-FGH, xylose-ABC transport. For other enzyme abbreviations refer to Fig. 5 legend. Data are derived from Gonzalez et al. (2002).

This is also the case in a glucose-limited chemostat where a microorganism is attempting to extract as much energy from the limited carbon to sustain growth (de Graef et al., 1999; Teixeira de Mattos & Tempest, 1983) or in an *E. coli* growing in minimal medium with xylose as C-source due to the low ATP yield of 0.67 per xylose to pyruvate utilizing the ABC transporter, XylFGH (Fig. 6) (Kim et al., 2007). An ATP yield of 0.67 mol/mol of xylose fermented is enough for maintenance of an *E. coli* culture in mineral salts medium and not enough to sustain growth. Acetate and ethanol production increases the anaerobic ATP yield to 1.5 per xylose to support growth in this medium (Hasona et al., 2004). However, as a culture approaches stationary phase, a decline in growth rate would dictate a reduction in ATP yield to balance ATP production with consumption (energy charge) (Atkinson, 1968). Lactate production becomes dominant under these conditions due to the lower ATP yield compared to acetate and ethanol as fermentation products (Kim et al., 2007). This shift in the product profile is in addition to reduction in sugar flux associated with lower growth rate as seen by lower specific productivity (Tao et al., 2001; Wang et al., 2015). In these instances, although the ATP yield per glucose is different, the product profile is governed by the energy charge while supporting redox balance.

**Fig. 7.**
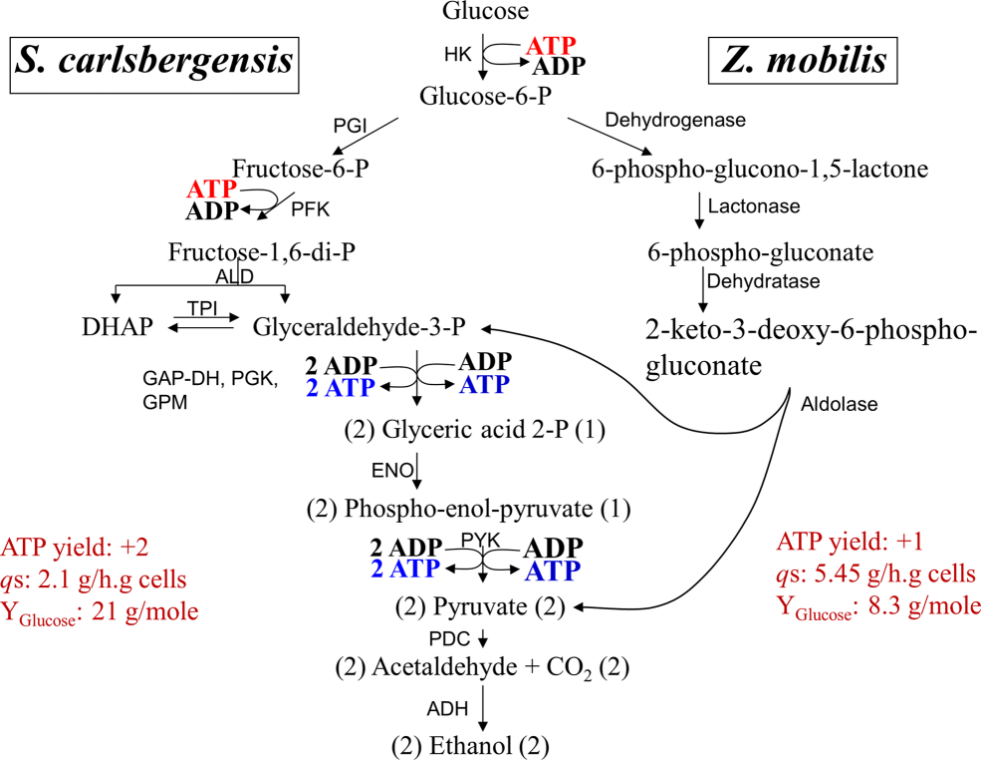
ATP yield and glucose flux in yeast and *Z. mobilis* during fermentation of glucose to ethanol. HK, hexokinase. For abbreviation of other enzymes refer to legends to Figs. 5 and 6. Data are derived from Rogers et al. (1979).

*Z. mobilis* uses Entner–Doudoroff pathway for glucose metabolism and this pathway yields only one net ATP per glucose fermented to two ethanols (Fig. 7) (Dawes et al., 1966). This lower ATP yield in *Z. mobilis* leads to an almost twofold higher glycolytic flux (qs) compared to yeast with an ATP yield of 2 per glucose (Rogers et al., 1979). This inverse correlation to net ATP yield and glycolytic flux to product (specific productivity) has also been reported for bacteria with *atp* mutations that affect intracellular ATP concentration (Koebmann, Westerhoff, Snoep, Nilsson, et al., 2002; Koebmann, Westerhoff, Snoep, Nilsson, et al., 2002).

In contrast to primary products, pathways leading to production of secondary products are generally not directly connected to ATP production or redox balance. The metabolic pathway to the designed product branches from the primary energy production pathways of aerobic or anaerobic metabolism. Classical examples of secondary products produced by bacteria and fungi are antibiotics, amino acids, etc. (Pham et al., 2019). Fatty acids, isoprenes, hydrocarbons, etc. are examples of secondary products with potential as energy sources (Dellomonaco et al., 2010; Kang & Lee, 2015; Liao et al., 2016; Zargar et al., 2017). Almost all the chemicals produced by a microbial biocatalyst that require O_2_ as an oxidant for redox balance can be considered as secondary metabolic products and are not further discussed in this communication.

## Optimizing Productivity

Specific and volumetric productivity are two important measures of biocatalyst potential with the latter being particularly significant for industrial production.

### Specific Productivity

**Fig. 8.**
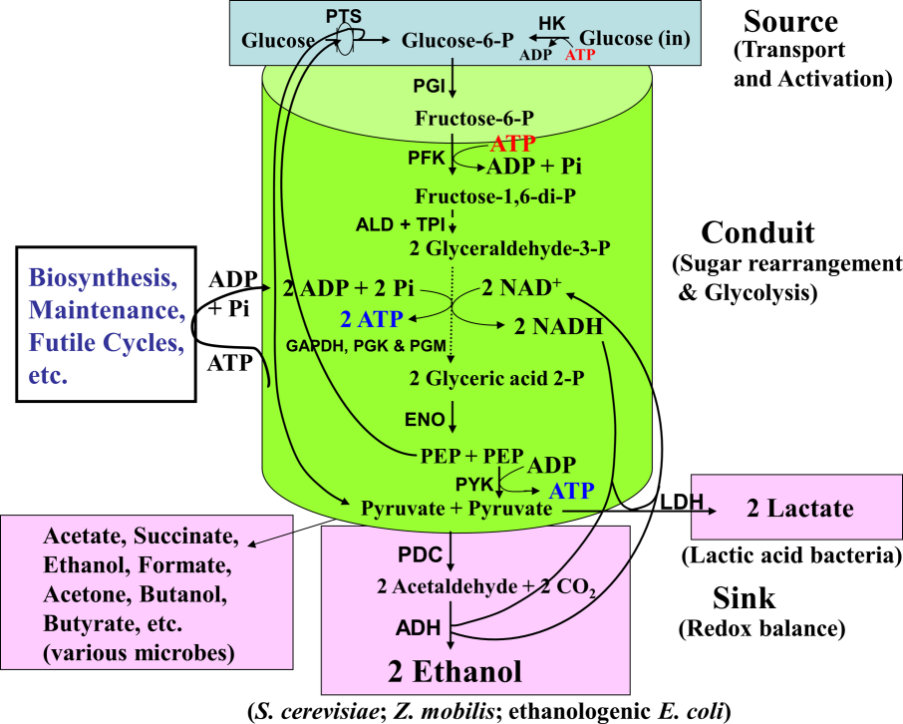
Three components of a sugar fermentation pathway leading to primary products. (1) Transport of sugars into the cell and its conversion to glucose-6-phosphate is termed as the “Source” since the transformation of sugar and glycolysis (EMP-pathway) starts from this sugar phosphate. (2) Conversion of glucose-6-phosphate to pyruvate (glycolysis) is termed the “Conduit.” (3) The “Sink” represents the direct reduction of pyruvate to lactate or products derived from pyruvate (ex. acetaldehyde to ethanol) to maintain redox balance during anaerobic growth. In nonethanologenic microbes, several other products could be produced for maintenance of redox balance (Fig. 5). Glucose can be replaced by other sugars and the appropriate form of these sugar phosphates can be considered as “Source.” Coupled reactions channel the ATP for growth and this must also be recycled to ADP for continued flow of carbon through the “Conduit.” For enzyme abbreviations, refer to Figs. 5–7 legends.

**Fig. 9.**
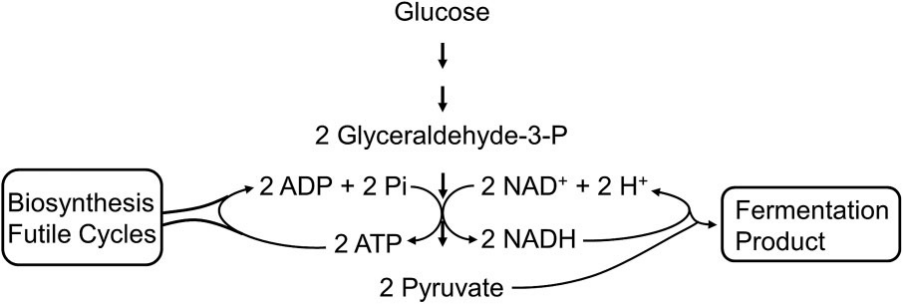
Coupled reactions required for support of glycolysis and product production.

**Fig. 10.**
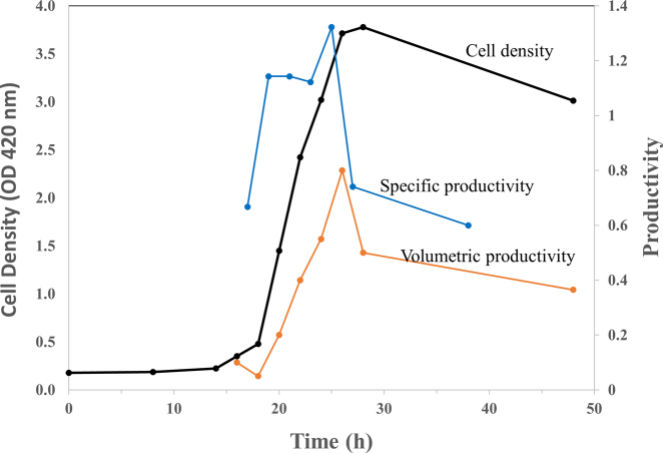
Volumetric and specific productivity of a primary product, butyrate, during various stages of growth of *Clostridium thermobutyricum*. Cell density is presented as optical density at 420 nm; specific productivity, g product/(g dry cell weight hr); volumetric productivity, g product/(l hr). Data derived from Wang et al. (2015).

Specific productivity is the rate at which a microbial biocatalyst converts sugars or other substrates to the desired product. During fermentation, the specific productivity is determined by the rate-limiting step in a set of coupled reactions that can be separated into three segments: entry of sugar into the cell and activation to sugar-phosphate (source), glycolysis (conduit), a set of reactions that convert the activated sugar to pyruvate while generating ATP and reductant, and conversion of pyruvate to product(s) (sink), such as ethanol to maintain redox balance (Fig. 8). These three parts of the overall process of converting sugar in the medium to primary fermentation product(s) need to be in synchrony for the highest rate of productivity and this is influenced by other coupled reactions such as ATP and NADH consumption. Due to the obligatory synthesis of ATP during glycolysis, the specific productivity directly correlates with ATP consumption to maintain a desired ratio of ATP/(ADP + AMP) (the adenylate charge; ([ATP]+½[ADP])/([ATP]+[ADP]+[AMP])). For optimum specific product productivity, the ATP production and consumption need to be in balance (Fig. 9) and an actively growing culture with the highest demand for ATP is expected to have the highest specific productivity relative to a culture that is reaching stationary phase or a nongrowing culture whose ATP requirement is modest (maintenance) in comparison to an actively growing culture (Fig. 10) (Tao et al., 2001; Wang et al., 2015). When ATP production is sub-optimal to the needs of the cell, as seen with an anaerobic growth mode, carbon flux to the product and thus the specific productivity increase to support that demand (Tempest & Neijssel, 1984). Therefore, one way of improving specific productivity of *E. coli* or yeast is to minimize ATP yield from the pathway either by using a glycraldehyde-3-phosphate oxidoreductase that does not couple to ATP production (Fourrat et al., 2007), arsenolysis to minimize net ATP yield (Welch & Scopes, 1985) or couple an ATP-futile cycle that lowers the adenylate charge below the optimum (Hadicke et al., 2015; Patnaik et al., 1992; Semkiv et al., 2016). It should be reiterated that a major fraction of glucose carbon is converted to product in an anaerobic ethanologenic microorganism and only a relatively small fraction (<5%; Martinez et al., 2007) ends up in biomass due to low net ATP yield of the overall process.

From a thermodynamic/kinetic point of view, the specific productivity is dependent on the ΔG (for our discussion here, ΔG°) of the overall reaction, glucose to product. The calculated ΔG° value for the conversion of medium glucose to 2 ethanols (+2 CO_2_) is −73.8 kJ/mol (Reaction 8) (Domalski, 1972). With co-generation of 2 ATPs per mole of glucose fermented, the ΔG° increase to −10.8 kJ/mol (Reaction 9). From the following relationship, —ΔG° = *RT*ln*K* and the temperature at 25°C and the gas constant (*R*) at 8.31 J/(deg mol), the rate constant of this overall reaction (*K* at standard reaction conditions) can be calculated to be 77.6.

(Reaction 8) \begin{eqnarray*} {{\rm{C}}_6}{{\rm{H}}_{12}}{{\rm{O }}_6} \to 2\,{{\rm{C}}_2}{{\rm{H}}_6}{\rm{O}} + 2\,{\rm{C}}{{\rm{O}}_2}\quad \Delta G^\circ &=& - 73.8\,{\rm{kJ/mol}};\quad\\ {\rm{K}} &=& 6.8 \times {10^{12}} \end{eqnarray*}

(Reaction 9) \begin{eqnarray*} {{\rm{C}}_6}{{\rm{H}}_{12}}{{\rm{O }}_6} &+& 2\,{\rm{ADP}} + 2\,{\rm{Pi}} \to 2{{\rm{C}}_2}{{\rm{H}}_6}{\rm{O}} + 2\,{\rm{C}}{{\rm{O}}_2} + 2\,{\rm{ATP}}\quad\\ && \Delta G^\circ = - 10.8\,{\rm{kJ/mol}};\quad {\rm{K}} = 77.6 \end{eqnarray*}

The *in vivo* rate of this process is certainly higher than this calculated rates to drive this reaction forward because of product removal (both ethanol and CO_2_) from the cytoplasm. By lowering the ATP yield during glucose fermentation from 2 to 1 as the case with *Z. mobilis*, the rate constant can be greatly improved with a higher glycolytic flux (Fig. 7) and specific productivity of ethanol (Reaction 10).

(Reaction 10) \begin{eqnarray*} &&{{\rm{C}}_6}{{\rm{H}}_{12}}{{\rm{O }}_6} + {\rm{ADP}} + {\rm{Pi}} \to {{\rm{C}}_2}{{\rm{H}}_6}{\rm{O}} + {\rm{C}}{{\rm{O}}_2} + ATP\quad\\ && \Delta G^\circ = - 42.3\,{\rm{kJ/mol}};\quad {\rm{K}} = 2.6 \times {10^7} \end{eqnarray*}

This also applies to hydrolysis of ATP to ADP and phosphate by a cytoplasmic ATPase or with a futile cycle, and release of energy as heat. Although lowering ATP yield per glucose increases specific productivity, it also decreases growth rate suggesting a need for achieving a balance between growth, cell density and productivity. This is discussed below.

Highest specific productivity during the early exponential phase of growth was also reported for *Z. mobilis* (Osman et al., 1987). At this time of highest specific productivity, the ethanol concentration in the medium reached to about 3.5% and continued to increase with growth and fermentation. The observed decline in specific productivity in this bacterium could be attributed to accumulation of product ethanol since the measured energy charge was not significantly altered during this phase of growth. This suggests the importance of identifying the potential toxicity of the fermentation product(s) from that of physiological limitations in attempts to improve specific productivity of engineered microbial biocatalysts.

As seen with ATP, NADH/NAD^+^ (redox balance) also plays a critical role in supporting high specific productivity and high yield (Figs. 8 and 9). Since NAD^+^ is a substrate for glyceraldehyde-3-phosphate dehydrogenase, its availability also modulates glycolytic flux. To maintain an optimum ratio of NADH/NAD^+^, the rate of conversion of pyruvate to desired product needs to be in synchrony with pyruvate production. These constraints also suggest that an anaerobic culture with a higher NADH/NAD^+^ is ideally suited for high specific productivity of primary products compared to aerobic mode of metabolism.

Since glycolysis in *E. coli* and yeast has evolved as an efficient anaerobic process (Melendez-Hevia et al., 1997), ATP flux and redox balance are the two main factors that dictate the specific productivity of product. Any attempt to increase primary product productivity needs to integrate these coupled reactions to the overall growth and physiology of the cell.

### Volumetric Productivity

Volumetric productivity of a product is determined by the specific productivity of the microbial biocatalyst and the cell concentration per unit volume. Since higher cell density leads to higher volumetric productivity at all specific productivity levels, it is important to optimize conditions to support high growth rate and cell yield (Underwood et al., 2002). As discussed above, the growth rate is directly proportional to the rate of ATP generation. Thus, aerobic cultures have an advantage over anaerobic cultures due to their higher growth rate and higher biomass yield (Y_glucose_) (Payne, 1970). A possible approach to take advantage of this is to start cultures with O_2_ to build the biomass rapidly and then switch to high cell density fermentation for high volumetric productivity. However, the specific productivity of such cultures may not be at the highest value, as slow growing (or nongrowing) high-density cultures transferred to fermentation conditions have low ATP requirement, like stationary phase anaerobic cultures. As an example, *Clostridium thermobutyricum* growing anaerobically and producing butyrate as a primary product, maintains a specific productivity of about 1.1–1.3 g/(g DCW hr) during the growth phase (Wang et al., 2015). During this phase of growth, volumetric productivity increases due to increasing cell density (Fig. 10). As the cells approach stationary phase of growth, a decrease of about 45% in specific productivity is matched by a drop of about 40% in volumetric productivity. The higher cell density at this stage is not compensating for the decrease in specific productivity.

One of the unanswered problems of industrial fermentation is sustaining high volumetric productivity of late-exponential growth phase cultures to stationary phase and to high cell density cultures for high product titers to lower residence time in industrial fermentations. One approach is to engineer the microbial biocatalyst for high ATP yield during growth phase and low ATP yield at the end of growth to support high rate of product production at high cell density. It has been demonstrated that functional ATP futile cycles (as well as ATPases) do increase productivity of cultures (Patnaik et al., 1992; Koebmann, Westerhoff, Snoep, Solem, et al., 2002). It might be possible to regulate net ATP yield through nonproductive ATP hydrolysis induced only after the culture reaches the desired high cell density. Low net ATP yield of such a culture could support high sugar flux to supply energy for maintenance without limiting productivity.

An alternate to futile cycles would be to remove pathways that produce ATP, such as the acetate kinase that generates one ATP per acetate produced. Fermentation of xylose to lactic acid yields only 0.67 ATP per xylose and due to this low yield, product productivities are higher with xylose as C-source compared to glucose (Hasona et al., 2004; Wang et al., 2019). In contrast, due to the low ATP yield, xylose may not be the best C-source for building biomass rapidly. Such a culture can be started with glucose for rapid build-up of biomass and then switched to xylose at the end of growth for fermentation. Another approach is to suspend the culture in a high specific productivity mode as in a chemostat. Unfortunately, continuous cultures tend to select for mutants on the basis of growth and not necessarily for product production and therefore are not industrially favored (Neijssel & Tempest, 1979).

Based on the discussion above, it is apparent that to maintain the highest productivity of a primary product it is prudent to start a culture with high net ATP yield to produce the highest amount of biomass rapidly followed by limiting net ATP yield during post growth phase for high specific and volumetric productivities. Microbial metabolic engineering incorporating the strategies discussed above can lead to a new generation of microbial biocatalysts for production of fuels and chemicals at high rates, yields, and productivities.

In the following sections, some of the principles discussed above are explored in the development of microbial biocatalysts for production of ethanol, as an example.

## Microbial Biocatalysts for Conversion of Sugars from Biomass to Ethanol

The yeast *S. cerevisiae* is the work horse in ethanol production and is used by the industry for fermentation of sucrose or glucose from grain starch to ethanol. Almost all industrially produced ethanol, about 16 billion gallons (>60 billion liters) in United States in 2018 (28.7 billion gallons, 108.6 billion liters, world-wide), is produced using this microorganism. This can be attributed to its optimized metabolic pathway from sugars to ethanol, in addition to having several other industrial advantages (Table 1). However, the years of evolution, selection and refinement that led to *S. cerevisiae* as an effective microbial biocatalyst for fermentation of glucose and sucrose to ethanol also made this microorganism ineffective in fermentation of pentose sugars derived from the hemicellulose fraction of lignocellulosic biomass as global ethanol industry shifts from food to nonfood carbohydrates as feedstock. Although *S. cerevisiae* has been engineered to ferment pentose sugars, the rate of fermentation of xylose by these derivatives is significantly lower than that of glucose (Demeke, Dietz, et al., 2013; Ko et al., 2016) indicating that the utilization of all biomass-derived sugars at high productivity, titer, and yield requires several additional characteristics in the microorganism (Table 1) in addition to the basic ability to ferment them. These required characteristics can be more readily identified and optimized in bacteria such as *E. coli* and later introduced into *S. cerevisiae* to improve fermentation of biomass-derived sugars in an industrial setting.

Several bacteria, including *E. coli*, ferment sugars in nonfood crops and many of these bacteria produce ethanol, but only as a minor product (Tables 3 and 4) (Clark, 1989; Ohta et al., 1991a, 1991b). This necessitated the need for a synthetic microorganism that can ferment all the sugars in nonfood carbohydrates (glucose, xylose, arabinose, galactose, mannose, etc.) completely to ethanol at high productivity and yield (Yomano et al., 2009). As discussed above, various components of the metabolic pathway (Fig. 8), source (sugar uptake and activation), conduit (conversion of the sugar-phosphate to pyruvate, ATP and NADH) and sink (oxidation of NADH for redox balance) in these engineered microorganisms need to be in balance for the highest glycolytic flux as in *S. cerevisiae* or *Z. mobilis*. Specific examples are listed below to illustrate this need.

**Table 4.** Source/Sink Relationship in Product Production by Ethanologenic *E. coli*

		Fermentation products (mM)					
Strain	*pdc/adh* ^a^	Ethanol	Acetate	Lactate	Succinate	Ethanol yield^b^ (g/g)	PDC activity (units)
Wild type	–	87	66	641	58	0.05	–
KO3	One	226	69	525	66	0.13	0.2
KO4	∼19 copies	1 148	22	29	70	0.56	2.1

*Note*. Cells were cultured in rich medium with glucose (100 g/l) and the concentration of fermentation products are from cultures at 72 hr at 37°C. Calculated ethanol yield of 0.56 for strain KO4, higher than the theoretical yield of ethanol from added glucose (0.51), is due to contribution of sugars from the medium components, tryptone, and yeast extract. PDC, pyruvate decarboxylase activity units are µmol/(min mg protein). Adapted from Ohta et al. (1991a, 1991b).

^a^Number of copies of *pdc* and *adh* genes are from Turner et al. (2012).

^b^Yield is expressed as g ethanol/g of added sugar consumed.

### Ethanologenic *E. coli*

An early example of synthetic biology for production of liquid fuels is ethanologenic *E. coli* (strain KO3). This strain contained two foreign genes; *pdc* and *adhB* encoding pyruvate decarboxylase (PDC) and alcohol dehydrogenase II (ADH-B), respectively, from *Z. mobilis* (Reaction 11) (Ohta et al., 1991a, 1991b).

**Figure:**
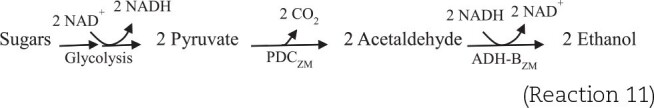


These two enzymes constituted a new ethanologenic pathway that increased the ethanol fraction in the fermentation products of *E. coli* (acetate, lactate, ethanol and succinate) (Table 4). PDC catalyzes decarboxylation of pyruvate to yield an electron acceptor acetaldehyde that is reduced to ethanol by ADH to maintain redox balance.

Although strain KO3 produced ethanol, this accounted for only ∼25% of the total fermentation products (Table 4) (Ohta et al., 1991a, 1991b) This low molar yield suggests that the introduced ethanologenic pathway (sink) is apparently operating at a lower rate than glycolysis in this strain (Fig. 8). Such an imbalance led to accumulation of pyruvate (and NADH) that is rapidly reduced to lactate to maintain redox balance. When the level of PDC and ADH were increased by about 10-fold (>2 units of activity; strain KO4; Table 4) (sink is enlarged), ethanol accounted for more than 90% of the total fermentation products along with a fourfold increase in volumetric productivity (Ohta et al., 1991a, 1991b). Both *E. coli* strains KO3 and KO4 used pyruvate and derivatives of pyruvate (acetaldehyde) as electron acceptors and the concentrations of the enzymes, PDC and ADH, are crucial to direct the carbon to ethanol and away from production of lactate and other fermentation products. Although strain KO11, a *frd* mutant of strain KO4, that lacks fumarate reductase and thus could not produce succinate as a fermentation product, fermented glucose to ethanol at almost 100% conversion efficiency when cultured in rich medium, the PDC and ADH levels were found to be limiting for ethanol productivity and growth when this strain was shifted to mineral salts medium to lower industrial production cost (Huerta-Beristain et al., 2008; Lawford & Rousseau, 1991). Increasing the expression level of *pdc* and *adhB* by promoter exchange and adaptive metabolic evolution, elevated growth rate, cell yield, and ethanol productivity in mineral salts medium compared to strain KO11 with xylose as carbon source (Yomano et al., 2008). These studies show the importance of integrating the introduced genes or pathways with cellular physiology to maintain the reductant sink (PDC and ADH in this case) at a high enough level for maximum productivity and yield. Furthermore, this reinforces the need for understanding the role of medium composition in cellular physiology and product productivity.

Native PTS-transport for glucose in *E. coli* appears to be sufficient for optimal rate of growth as seen with Pts^−^ mutants with alternate glucose transporters (Galactose permease or *Z. mobilis* glucose facilitator and glucokinase) (Hernandez-Montalvo et al., 2003; Snoep et al., 1994). Replacing PTS with elevated expression of galactose permease (GalP) and glucose kinase (Glk) restored normal growth rate and *q*_glucose_ at a glucose transport rate of about 60% of the native PTS. Even at this reduced glucose transport, specific ethanol productivity of engineered *E. coli* was about twofold higher during the growth phase, compared to a PTS^+^ strain, suggesting that the known regulatory aspects of PTS system (Deutscher et al., 2006) play a critical role in overall rate of glucose conversion to product. Due to the complex control of glycolytic pathway, overexpression of glycolytic enzymes, either singly or in combination, appears to have minimal effect on ethanol productivity in growing cells of *S. cerevisiae* or ethanologenic *E. coli* (Jojima & Inui, 2015).

It appears that optimizing the enzyme activity at the “Sink” level and integrating this with the other two parts of the overall pathway is critical for high product productivity.

## Carbon Partitioning During High Rates of Ethanol Production

A high ethanol titer is required in an industrial setting to minimize the cost of distillation (Kvaalen et al., 1984) and the overall economy of the biorefinery. Overzealous expression of the “sink” proteins to maximize productivity can deprive the cells of the needed carbon skeletons for biosynthesis and other cellular functions, especially in mineral salts medium and such a culture is not expected to reach the desired biomass level to support the intended high volumetric productivity. Thus, an optimal microbial biocatalyst needs to appropriately distribute the carbon between cell mass (biosynthesis; the main ATP sink) and the product (to oxidize the NADH). An imbalance favoring one or the other can have negative effect on productivity due to lower growth rate and cell mass. Adaptive metabolic evolution for growth of the microbial biocatalyst with appropriate selection normally identifies specific cells with proper balance between growth and product productivity.

In addition to the need for supporting biomass production, carbon partitioning is also important during sugar fermentation at high concentrations (>100 g/l). High sugar concentrations can induce osmotic stress that limit productivity due to lower growth rate unless the cell compensates for this by producing osmoprotectants. Osmotic stress is introduced by an imbalance in water activity across the cell membrane by high solute concentration in the immediate vicinity of the cell membrane compared to the cytoplasm. If the sugar is rapidly consumed by the fermenting cell at a rate that is higher than the rate of diffusion to the cell membrane surface, the osmotic stress exerted by that sugar in the bulk medium could be minimal. This suggests that a sugar that is rapidly fermented, such as glucose, will exert less of an osmotic stress on the cell than a sugar that is fermented at a lower rate, such as xylose, at the same molar concentration (Miller & Ingram, 2007). There are several chemicals produced by the cell that help protect against hyperosmotic stress and all of these require appropriate partitioning of carbon at the specific branch point between the desired product and needed osmoprotectant.

As an example, xylose at 90 g/l (0.6 M) exerts osmotic stress on ethanologenic *E. coli* (Underwood et al., 2004) due to the high rate of ethanol production (“sink” activity; Fig. 8) that diverts almost all of the pyruvate to ethanol and away from glutamate, a needed osmoprotectant. The osmotic stress in these cultures was minimized by supplementing the medium with pyruvate that increased the cytoplasmic pyruvate level by about fourfold, to additionally supply carbon for glutamate production (Underwood, Zhou, et al., 2002). Other genetic and physiological modifications that increased the glutamate concentration in the cell also minimized the osmotic stress induced by high xylose concentration (Underwood et al., 2004). An unintended effect of osmotic stress induced growth inhibition is an increase in the NAD(P)H/NAD(P)^+^ ratio in the cell. Since NADH is an allosteric inhibitor of citrate synthase, the key enzyme leading to glutamate production, this increase in NADH pool is an additional factor that also contributes to osmotic stress mediated lower growth rate. This is supported by the fact that replacing the native *E. coli* citrate synthase with a *B. subtilis* homolog that is insensitive to allosteric inhibition by NADH restored normal growth and fermentation in the presence of high xylose concentration (Underwood et al., 2002). These studies show that the osmotic stress induced by high sugar concentrations can be minimized without compromising productivity by judicious engineering of the microbial biocatalyst so that carbon is appropriately distributed at the pyruvate node.

The physiological information gained during the development of an effective ethanologenic *E. coli* is readily applicable to other microbial biocatalysts designed for production of other biofuels. The main difference between ethanologenic and other microbial biocatalysts is the change of the “Sink” from ethanol to butanol or other primary product of choice. Adaptive metabolic evolution of the synthetic microbial biocatalyst for high productivity and titer is an essential component in the development process of an effective microbial biocatalyst as this allows the selection of “best fit” for biofuel production. Physiological insights from genome scale analysis of these adapted microbial biocatalysts can be combined in the development of microbial biocatalysts with higher productivity.

## Partitioning Reductant During Inhibitor Tolerance

Lignocellulosic biomass consists of ∼25% hemicellulose, that can be hydrolyzed to free sugars and other products by a set of enzymes (xylanases, xylosidases, arabinofuranosidases, esterases, and α-glucuronidases) or by mild acid treatment at elevated temperatures (Falls et al., 2011; Geddes et al., 2010; Tao et al., 2011). Acid treatment, although relatively easy and low cost, does release microbial growth inhibitors (Fig. 2) (Martin et al., 2018). Among these inhibitors generated during acid hydrolysis of biomass, furfural, a dehydration product of xylose, is the most abundant and furfural is also known to enhance the effect of other minor inhibitors in the biomass acid hydrolysate (Shi et al., 2016). The mechanism of microbial growth inhibition by furfural is apparently related to the ability of this aldehyde to create single strand breaks in DNA in addition to its effect on undetermined hydrophobic targets in the cell (Mills et al., 2009; Zaldivar et al., 1999). Due to the direct effect of furfural on cellular components and its ability to traverse the cell membrane owing to its hydrophobicity, toxicity of furfural and other related aldehydes needs to be significantly reduced, either chemically or biologically, to support growth of microorganisms. Since an engineered microbial biocatalyst designed for fermentation of sugars derived from biomass is expected to function optimally in the presence of these inhibitors, both yeast and ethanologenic *E. coli* are being adapted to grow in the presence of acid hydrolysis derived inhibitors (Demeke, Dumortier, et al., 2013; Geddes et al., 2015; Miller et al., 2009; Shi et al., 2016; Wang, Miller et al., 2011).

### Competition between Biosynthesis and Furfural Tolerance

Tolerance to furfural is derived from microbial reduction of the aldehyde utilizing NADPH as the reductant to furfuryl alcohol, a relatively less toxic compound compared to furfural (Gutierrez et al., 2002, 2006). However, high-affinity furfural reductases such as YqhD in *E. coli* appear to deplete the cellular NADPH pool that limits growth of the microbial biocatalyst until most of the furfural is removed from the medium. Addition of sulfur-containing amino acids to the medium with furfural minimizes the growth-inhibitory effect of this inhibitor (Miller et al., 2009, 2010; Turner et al., 2011). This suggests that diversion of NADPH to reduce furfural in ethanologenic *E. coli* leads to starvation of NADPH-dependent biosynthesis, particularly sulfur-containing amino acids. In a fermenting microbial biocatalyst, most of the NADPH is generated from NADH, the primary reductant produced by glycolysis, by transhydrogenases, on demand. Apparently, the rate of production of NADPH from NADH is not high enough to support both biosynthesis and reduction of furfural and similar inhibitory compounds in the medium. Replacing NADPH-dependent YqhD with an NADH-dependent nonspecific aldehyde reductase FucO (1,2-propanediol oxidoreductase) in which NADH is used for reduction of furfural and NADPH is used for biosynthesis reduced the severity of this inhibition (Wang, Miller, et al., 2011). These studies show that in addition to source/conduit/sink optimization (Fig. 8), competing reactions for cofactor and the coupled reactions that generate the specific reductant also impart significant influence in product productivity by influencing microbial growth.

## Simultaneous Consumption of Two or More Sugars

Lignocellulosic biomass contains two major polysaccharides, cellulose, a glucose polymer, and hemicellulose, a mixture of hexoses and pentoses. Since all microorganisms have a preference for a specific C-compound as an energy source, for example, glucose in *E. coli*, utilization of a second C-source(s) starts only after the preferred sugar is exhausted from the medium or is growth rate-limiting in a medium containing two or more C-sources. Almost all secondary sugars require one or more enzyme-catalyzed steps before the metabolic intermediate can enter the EMP-pathway and production of these enzymes, including transport, accounts for the observed lag before consumption of the second sugar commences (Diauxie) (Ullmann, 2010). In addition, glucose is also known to negatively impact transport of many of the secondary sugars (inducer exclusion) (Farwick et al., 2014; Saier, 1998). Diauxic growth of *E. coli* in a medium containing glucose and lactose is a classic example of this preference and this has been extended to other sugars including xylose (Hasona et al., 2004; Monod, 1966). The mechanism of catabolite repression (CCR) in microorganisms is well studied and is not discussed here (Magasanik, 1961; Saier, 1998).

The lag observed during sequential utilization of glucose and xylose in *E. coli* or in a xylose-fermenting yeast is of concern in industrial fermentation of sugars from lignocellulosic biomass as nonproductive due to an increase in operating time and the presence of residual sugars in the spent medium. There is a growing interest in engineering microorganisms for simultaneous utilization of multiple sugars to eliminate this lag for rapid fermentation of a mixture of sugars to products (Gao et al., 2019; Kim et al., 2010; Wu et al., 2016). In the following section, discussion is limited to glucose and xylose, the two major sugars of plant biomass, for brevity. Several strategies have been employed to achieve the goal of simultaneous utilization of multiple sugars by microbial biocatalysts (Wu et al., 2016). Invariably, these studies lowered the rate of glucose consumption at the level of transport (Dien et al., 2002; Lane et al., 2018). Glucose utilization in *E. coli pts* mutants was restored by GalP for glucose transport and phosphorylation of glucose by glucokinase. In these mutants also, xylose utilization started only when the glucose concentration in the medium decreased below a critical level (Balderas-Hernandez et al., 2011). However, ethanol production from a mixture of glucose and xylose did not exhibit a lag that was seen with the wild type. Coutilization of xylose with glucose in these *E. coli* mutants is apparently due to reduced catabolite repression of xylose consumption in the presence of glucose, a result of removal of a key component of CCR in *E. coli*, the PTS system (Gosset, 2005; Kim et al., 2010). It should be noted that in these and other similar mutants, the specific rate of consumption of glucose alone or glucose and xylose together (glycolytic flux) were comparable (Balderas-Hernandez et al., 2011; Roca et al., 2004).

These results suggest that glucose by itself supports the highest glycolytic flux in ethanologenic *E. coli* and yeast (saturating the “conduit”; Fig. 8). Increasing the pool of glycolytic intermediates from a side pathway such as xylose metabolism (fructose-6-phosphate and glyceraldehydehyde-3-phosphate) (Fig. 6), may not further increase the glycolytic flux due to the complex regulation of the key enzymes of the pathway and the control of the overall flux by coupled reactions (see previous sections). A consequence of this is to physiologically limit xylose utilization until carbon flow through the “Conduit” is below the maximum capacity when the medium concentration of glucose is below the needed optimum (Fig. 8). Co-utilization of xylose (or other sugars) even in the presence of high concentration of glucose would require an increase in the overall glycolytic flux (enlarging the “conduit”; Fig. 8) of a glucose-fermenting microorganism to accommodate this increase in pool levels of xylose-derived glycolysis intermediates. Since glycolytic flux is controlled by various coupled reactions, especially energy consumption, a complex set of reactions need to be balanced to accommodate the higher glycolytic flux in a growing cell. With a reduction in glucose transport, either by a mutation (*pts*) or lower medium concentration, glycolytic flux is apparently operating at a lower than the maximum achievable level and xylose provides the additional glycolytic intermediates to maximize glycolytic flux.

An alternate method to eliminate the lag before xylose utilization starts in a medium containing both glucose and xylose is to increase the rate of conversion of pyruvate to products as seen with ethanologenic *E. coli* strains by increasing the level of PDC/ADH (enhanced “Sink”; Fig. 8) that is pulling the carbon faster to ethanol (Shanmugam et al., 2020). Rapid removal of pyruvate due to its conversion to ethanol (strain KO11) and decreasing glucose concentration in the medium can allow the xylose utilization pathways to be induced and active to support optimal rate of glycolysis. Under this physiological condition, a seamless transition from glucose to xylose fermentation can be achieved.

Methylglyoxal is a product of imbalance between the upper (glucose to 2 triose-phosphates) and lower (glyceraldehyde-3-phosphate to pyruvate) parts of glycolysis and is derived from dihydroxyacetone-phosphate (DHAP). This imbalance can lead to accumulation of DHAP and methylglyoxal synthase (encoded by *mgsA*) acts as a valve to remove the excess DHAP to optimize overall glycolytic flux (Weber et al., 2005). The toxic methylglyoxal is rapidly converted to lactate and excreted into the medium. A mutation in *mgsA* is expected to close this alternate pathway and force DHAP-carbon to flow through the lower part of glycolysis to pyruvate, necessitating higher glycolytic flux. Such an increase in volumetric productivity was reported for *mgsA* mutants of ethanologenic *E. coli* strain in comparison with the corresponding parent (Yomano et al., 2009). This higher ethanol productivity also supported co-fermentation of multiple sugars in the medium. The physiological and regulatory mechanism leading to this increase in productivity needs exploration.

Attempts to increase the level of expression of the enzymes needed for utilization of the secondary sugars independent of catabolite repression without physiological accommodation of the higher influx of glycolytic intermediates has the potential for growth-inhibitory phenotype due to production of methylglyoxal and/or sugar-phosphate (Ackerman et al., 1974; Fraenkel, 1968; Kadner et al., 1992; Richards et al., 2013). These studies show the complex nature of the primary energy production pathways and associated coupled reactions, and the need for a better understanding of the physiological implications for successful metabolic engineering of ethanologens for simultaneous utilization of multiple sugars.

## Other Chemicals

*E. coli* and other microorganisms are being engineered to produce several other products with potential interest as a source of fuel, such as butanol, longer chain alcohols, isoprenes, alkanes, alkenes, fatty acids, fatty alcohols, methylketones, etc. (Atsumi et al., 2008; Choi & Lee, 2013; Dellomonaco et al., 2011; Fatma et al., 2018; Goh et al., 2012; Kung et al., 2012; Lennen & Pfleger, 2013; Li et al., 2012; Machado et al., 2012; Shen et al., 2011). Constructions of microbial biocatalysts for production of these chemicals are excellent examples of synthetic biology and metabolic engineering. However, low titers and yields of these products are physiological challenges that need to be addressed before reaching cost-effective commercial production. Several reviews discuss various engineering strategies for production of these chemicals and the readers are referred to these (Choi & Lee, 2013; Dellomonaco et al., 2011; Li et al., 2010, 2012; Liu & Khosla, 2010; Peralta-Yahya et al., 2012).

World-wide research on metabolic engineering of various microorganisms is expected to yield a collection of microbial biocatalysts that can also function in the presence of inhibitory compounds, such as furfural and others. In parallel, a new set of energy crops and process conditions are being developed to provide the needed sugars for fermentation. The future is bright for this nascent bio-based fuel and chemical industry that will rely heavily on microorganisms at every stage of this overall process.

## Other Compounds as Feedstocks for Production of Fuels and Chemicals

### Lignin

Lignin, a complex, aromatic, highly branched, substituted, hetero polymer, is an abundant energy resource in the biosphere accounting for about 20–30% of biomass. Although this polymer has a higher energy density compared to carbohydrates (23–26 MJ/ton compared to 18.6 MJ/ton for carbohydrates) (Baker, 1983), it is rarely used as a feedstock for production of fuels due to challenges in microbial depolymerization, conversion of the resulting monomers to central metabolism intermediates and toxicity of the monomers to many microorganisms (Abdelaziz et al., 2016; Beckham et al., 2016). As a result, lignin is generally burned for energy and heat and has not reached its potential as a feedstock for bulk chemicals and fuels.

Better understanding of enzymatic depolymerization of lignin and identification of anaerobic microbial processes for conversion of the monomers to central metabolism intermediates such as acetyl-CoA, pyruvate and succinyl-CoA have opened new opportunities for this neglected resource (Abdelaziz et al., 2016). Even with these developments in biochemistry, microbiology and synthetic biology, the higher oxidation state of carbon in lignin monomer [average structure of (CH_1.1_O_0_._35_)_10_], compared to sugars (CH_2_O)*_n_* would dictate a lower yield or require input of a reductant such as H_2_ in the production of energy rich fuel molecules, like ethanol, (CH_3_O_0.5_)_2_ making lignin unattractive for fuel production. However, lignin can and continues to serve as a source of various fine chemicals and aromatics including vanillin (Abdelaziz et al., 2016).

### Glycerol

Another chemical that is expected to be generated in large quantities with an increase in biodiesel production is glycerol. In their pioneering work, Gonzalez and his coworkers have developed several strategies for converting glycerol to various chemicals and fuels (Clomburg & Gonzalez, 2010, 2013). Since glycerol carbon is more reduced (CH_2.67_O) than glucose (CH_2_O), the additional reductant can be used for synthesis of other primary products of fermentation that are more reduced than ethanol, such as butanol, at close to the theoretical yield (Reaction 12) without the redox balance mandated co-products, such as acetone (Table 3) (Chen et al., 2019). The additional H_2_ can serve as a fuel or as a reductant for production of other chemicals including those originating from feedstocks such as lignin in a biorefinery that is co-producing multiple products.

(Reaction 12) \begin{eqnarray*} 2\,{{\rm{C}}_3}{{\rm{H}}_8}{{\rm{O }}_3} \to {{\rm{C}}_4}{{\rm{H}}_{10}}{\rm{O}} + 2\,{\rm{C}}{{\rm{O}}_2} + 2\,{{\rm{H}}_2} + {{\rm{H}}_2}{\rm{O}} \end{eqnarray*}

### Methane

Although methane, a highly reduced form of C, is a fossil-derived energy source, modern technology has tapped into vast reservoirs of this chemical in certain regions of the world (USA, Russia, etc.) where supply overwhelms demand. Significant amount of methane that has a greater global warming potential than CO_2_ is also produced biologically (anaerobic digestion) (Montzka et al., 2011). Although this gas can be used directly as a transportation fuel, there is increasing interest to upgrade methane to various liquid fuels and chemicals (gas to liquid; GTL) (Fei et al., 2014; Haynes & Gonzalez, 2014). Microorganisms are actively engineered for production of various platform chemicals with methane as the feedstock (methanol, formaldehyde, etc.) for further conversion to a desired final product. Aerobic microorganisms dominate the studies on upgrading methane while anaerobic microorganisms are being recognized in this process. However, low rates of productivity impede the development of industrially relevant biological processes utilizing methane as the feedstock. Synthetic methylotrophs, currently under construction are expected to improve the low productivity (Bennett et al., 2018). Coupling a chemical process that generates a triose, dihydroxyacetone, from methane using chemical catalysts with a microbial fermentation of this sugar to a desired product (Wang et al., 2018) has the potential to overcome the rate-limiting reactions in the conversion of methane to fuels and chemicals.

## Aerobic or Anaerobic Process for Production of Fuels and Chemicals

**Fig. 11.**
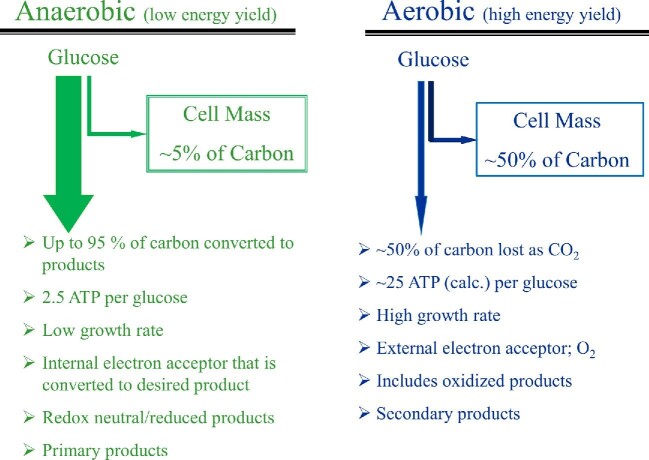
Overview of the physiological characteristics of aerobic and anaerobic metabolism of microbial biocatalysts.

As discussed above, primary products of fermentation are derived in an anaerobic or O_2_-limited environment while aerobic metabolism is utilized for production of several other chemicals (Dawes et al., 1966; Yomano et al., 2008; Liao et al., 2016). An overview of the two metabolisms is presented in Fig. 11. In addition to the physiological advantages of anaerobic mode (except the lower growth rate) that also conserves significant amount of energy in starting sugars in products, cost and other problems associated with aeration need to be considered in the choice of production mode. These include higher process cost associated with pumping, mixing and logistics of maintaining O_2_ at the desired level. It should be noted that the important parameter is not the dissolved O_2_ concentration at bulk liquid (measured at the probe surface) but the concentration of O_2_ at each cell surface and this needs to exceed the rate of consumption (respiration) by the microorganism. Due to the difficulty of maintaining uniform dissolved O_2_ concentration in large industrial tanks, localized anaerobic/O*_2_*-limited microenvironments develop leading to fermentation-derived co-products. These co-products, such as acetate, in addition to contaminating the product of choice, also has the potential to inhibit growth and productivity of the microbial biocatalyst. It is anticipated that metabolic engineering strategies for production of all bulk chemicals, including alternate fuel molecules, will include the advantages of anaerobic industrial fermentation to minimize cost of production of the desired chemical at the highest productivity and yield.

## Conclusion

Microorganisms play critical roles in the global biogeochemical cycles. They are involved in our energy cycles by supporting plant growth and thus biomass production, by degrading biomass to sugars through enzyme hydrolysis and by fermenting sugars to products. Based on the principles derived by Darwin more than 150 years ago, the primary metabolic activity of all living systems is to support the highest rate of growth of the organism in the specific environment, and thus, it stands to reason that in a closed anaerobic system, the highest productivity would be for the terminal product of this metabolic activity. Production of potential fuels, such as ethanol, is dependent on a balanced redox state, without which high rates of growth and productivity are not possible. An understanding of the innate physiological principles of microbial energy production/consumption and redox balance is crucial for optimal metabolic engineering of appropriate microbial biocatalysts to extend the catalog of fermentation products that can serve as liquid transportation fuels. The material presented here is intended to stimulate our thinking toward development of improved biocatalysts for cellulosic ethanol that can withstand the inhibitors generated during acid hydrolysis of biomass, ferment the multitude of sugars derived from the biomass efficiently without product inhibition and toxicity and also producing glycan hydrolases that can lower or eliminate the need for commercial enzymes, preferably using fermentation reactions rather than aerobic processes. The design of these microbial biocatalysts would be based on the physiological requirements described above and will employ the tools of synthetic and systems biology with the aim to produce next generation bio-based fuels and chemicals at commercially viable productivity, titer, and yield.
